# Return-to-Work and Early Functional Recovery Following Office-Based Ultrasound-Guided Microinvasive Carpal Tunnel Release: A Prospective Cohort Study

**DOI:** 10.3390/medicina62081441

**Published:** 2026-07-24

**Authors:** Christian A. Lobos, Kyle Wilcox, Aidan Cottrell, Thomaz De Campos Silva, Shea Wilcox

**Affiliations:** 1 Carpal Tunnel Institute, Melbourne, VIC 3056, Australia; 2 Carpal Tunnel Australia, Adelaide, SA 5000, Australia; 3 Carpal Tunnel Australia, Brisbane, QLD 4000, Australia; 4 Carpal Tunnel Australia, Canberra, ACT 2600, Australia; 5 Carpal Tunnel Australia, Sydney, NSW 2000, Australia; 6 Faculty of Health, Torrens University, Melbourne, VIC 3000, Australia

**Keywords:** carpal tunnel syndrome, ultrasound-guided surgery, microinvasive release, return to work, functional recovery, Boston Carpal Tunnel Questionnaire

## Abstract

*Background and Objectives*: Carpal tunnel release (CTR) has evolved toward increasingly less invasive techniques, with the aim of reducing procedural trauma and accelerating recovery. While ultrasound-guided and microinvasive approaches have demonstrated safety and technical precision, prospective data describing Return-to-Work (RTW) and early functional recovery remain limited. *Materials and Methods*: This prospective cohort study included adults undergoing office-based ultrasound-guided microinvasive CTR (Micro-CTR) under local anaesthesia using a retractable needle-mounted blade system. Patient-reported outcomes were collected using the Comprehensive Carpal Tunnel Recovery Questionnaire. The primary outcome was time to RTW. Secondary outcomes included change in pain score, analgesic duration, and time to return to driving and household tasks. Continuous outcomes were non-normally distributed and are reported as medians with interquartile ranges (IQRs). Recovery outcomes were compared by structured self-reported postoperative-event status using non-parametric and time-to-event analyses, and an exploratory distributional analysis assessed the influence of longer self-reported RTW intervals on the overall recovery distribution. *Results*: Sixty-seven patients were included. Median RTW was 9 days (IQR, 30.5; *n* = 48), with a median return to driving at 5 days (IQR 6) and household tasks at 7 days (IQR 7). Median pain decreased from 8/10 preoperatively to 0/10 postoperatively (*p* < 0.0001). Participants reporting at least one structured postoperative event had longer RTW intervals than those selecting no structured event (median 35 vs. 5 days; HR 0.42, 95% CI 0.22–0.80; *p* = 0.0079). Exploratory distributional analysis showed that longer RTW intervals had a substantial influence on the overall RTW distribution; the full RTW responder cohort remained the primary analysis. *Conclusions*: Ultrasound-guided Micro-CTR was associated with rapid early functional recovery in most patients in this prospective cohort. Recovery was heterogeneous, with longer RTW intervals observed among participants reporting structured postoperative events. These findings support RTW as a pragmatic functional endpoint in evaluating emerging CTR techniques.

## 1. Introduction

Carpal tunnel syndrome (CTS) is the most common entrapment neuropathy of the upper limb, affecting approximately 3–5% of adults [1] and contributing substantially to work-related disability [2], healthcare utilisation [3], and productivity loss [4,5,6]. Carpal tunnel release (CTR) remains the definitive intervention for moderate-to-severe or refractory disease and has demonstrated durable efficacy across multiple modalities [7,8,9].

Traditional measures of success following CTR have focused on symptom relief, grip strength, and neurophysiological improvement [7,8]. However, these outcomes do not fully capture the functional and socioeconomic consequences of CTS, particularly in working-age patients. Return-to-Work (RTW) has therefore emerged as a pragmatic endpoint that reflects not only clinical recovery but also occupational reintegration and broader functional restoration [5,10,11].

Historically, open CTR was the operative standard, with typical RTW intervals often measured in weeks because of wound-related discomfort and soft-tissue morbidity [12,13,14,15]. Endoscopic techniques subsequently reduced incision size and tissue disruption, with corresponding reductions in recovery times while maintaining decompression efficacy [16,17,18,19,20,21]. More recently, ultrasound-guided CTR has further advanced procedural visualisation by enabling real-time identification of the transverse carpal ligament (TCL) and other adjacent neurovascular structures [22,23]. Clinical studies have reported favourable safety profiles, durable outcomes, and shortened recovery intervals, with median RTW commonly reported between 10 and 21 days [24,25,26,27].

Ultraminimally invasive and Micro-CTR techniques represent a further refinement of this trajectory. These approaches use ultrasound guidance with needle-mounted blade, hook-knife, or thread-based systems to achieve ligament release through puncture-sized access under local anaesthesia. Thread-based approaches were first described as a non-scalpel technique using percutaneously looped thread transection of the TCL [28], and subsequent anatomical studies have continued to evaluate the feasibility, completeness of release, and safety of ultrasound-guided thread release in cadaveric models [29]. In a recent cadaveric study of ultrasound-guided thread release, complete TCL transection was achieved in 11 of 15 specimens, with no neural or vascular injury, although ultrasound visibility varied and incomplete transection occurred in four cases [29]. These findings support the broader technical feasibility of ultrasound-guided percutaneous release while also highlighting the importance of operator expertise, anatomical visualisation, and technique-specific outcome reporting.

Early cadaveric and clinical studies of retractable needle-mounted blade systems have demonstrated complete TCL division with mean RTW intervals in the range of 5–10 days [30,31,32,33]. Such findings suggest that reduced procedural invasiveness may be associated with earlier functional recovery, although outcome reporting remains heterogeneous, and device-specific evidence should not be treated as interchangeable.

That heterogeneity is an important limitation in the current literature. RTW reporting varies considerably across studies because of differences in study design, occupation type, compensation status, psychosocial context, and outcome definitions [34,35,36]. Our recent narrative review of RTW across CTR modalities highlighted this variability while also identifying a consistent overall trend toward shorter recovery intervals with less invasive techniques [36]. These observations underscore the need for prospective cohort data using a standardised, patient-reported functional recovery measure.

The present study evaluates patient-reported RTW and early recovery milestones following office-based Micro-CTR using percutaneous access. The aim was to provide prospective cohort data describing early functional recovery following this microinvasive technique while exploring factors associated with delayed RTW, without presupposing comparative superiority over other CTR modalities.

The primary aim was to quantify RTW intervals following office-based Micro-CTR performed under local anaesthesia. Secondary aims were to evaluate early recovery milestones, including pain reduction, analgesic duration, return to driving, and return to household tasks, and to explore clinical and recovery-related factors associated with delayed RTW.

We hypothesised that Micro-CTR would be associated with early occupational reintegration within the range of previously reported outcomes for less invasive CTR techniques. We further anticipated that postoperative pain, analgesic duration, compensation status, and complication profile would be associated with RTW variability.

## 2. Materials and Methods

### 2.1. Study Design and Setting

This study was designed as a prospective observational cohort investigation conducted at a single centre. The study evaluated patient-reported recovery and functional reintegration following office-based Micro-CTR using a percutaneous access system. Data for the analysed cohort were collected between 25 November 2025 and 1 March 2026. All participants provided written informed consent prior to enrolment. Reporting was guided by the Strengthening the Reporting of Observational Studies in Epidemiology (STROBE) statement [37].

### 2.2. Participants

Eligible participants were adults aged ≥18 years with clinically and electrodiagnostically confirmed CTS [38,39] who underwent ultrasound-guided Micro-CTR under local anaesthesia and completed both preoperative and postoperative questionnaires. Patients were included if they were employed or otherwise engaged in work or functional activities permitting assessment of RTW or recovery milestones.

Patients were excluded if they had undergone revision CTR [40], had neuropathies other than CTS, rheumatoid arthritis, or pregnancy, or had incomplete survey data or withdrawn consent.

### 2.3. Pre-Procedure Ultrasound Assessment

Prior to ligament division, all participants underwent ultrasound assessment of the carpal tunnel and adjacent structures as part of the procedural planning. The transverse carpal ligament, median nerve, ulnar neurovascular bundle, superficial palmar arch, flexor tendons, and relevant soft-tissue planes were assessed under high-frequency ultrasound. Particular attention was given to the anatomical features that could affect procedural safety or feasibility, including median nerve position, vascular structures, and local soft-tissue anatomy. Ultrasound assessment was used to confirm a safe procedural corridor and guide real-time planning of the access trajectory.

The study did not include formal sonographic outcome measures such as quantitative median nerve cross-sectional area, postoperative ultrasound scoring, or independent imaging confirmation as a research endpoint. Accordingly, ultrasound was used for procedural planning and real-time guidance rather than as a separate imaging outcome domain.

### 2.4. Surgical Technique

All procedures were performed using a standardised ultrasound-guided Micro-CTR protocol developed from the technique described by Hebbard et al. [30,31]. Procedures were completed in an outpatient office-based setting under strict aseptic conditions and entirely under real-time ultrasound visualisation using a FUJIFILM Sonosite PX ultrasound system (FUJIFILM Sonosite, Inc., Bothell, WA, USA) with a high-frequency L15-4 linear transducer (4–15 MHz).

Patients were positioned supine with the arm abducted and the forearm supinated on a draped hand table. The wrist was supported in gentle dorsiflexion. Following skin preparation with 2% chlorhexidine, the operative site was draped using a sterile carpal tunnel pack. The ultrasound transducer was covered with a sterile probe sheath, and sterile ultrasound gel was used to optimise acoustic coupling.

**Figure 1:**
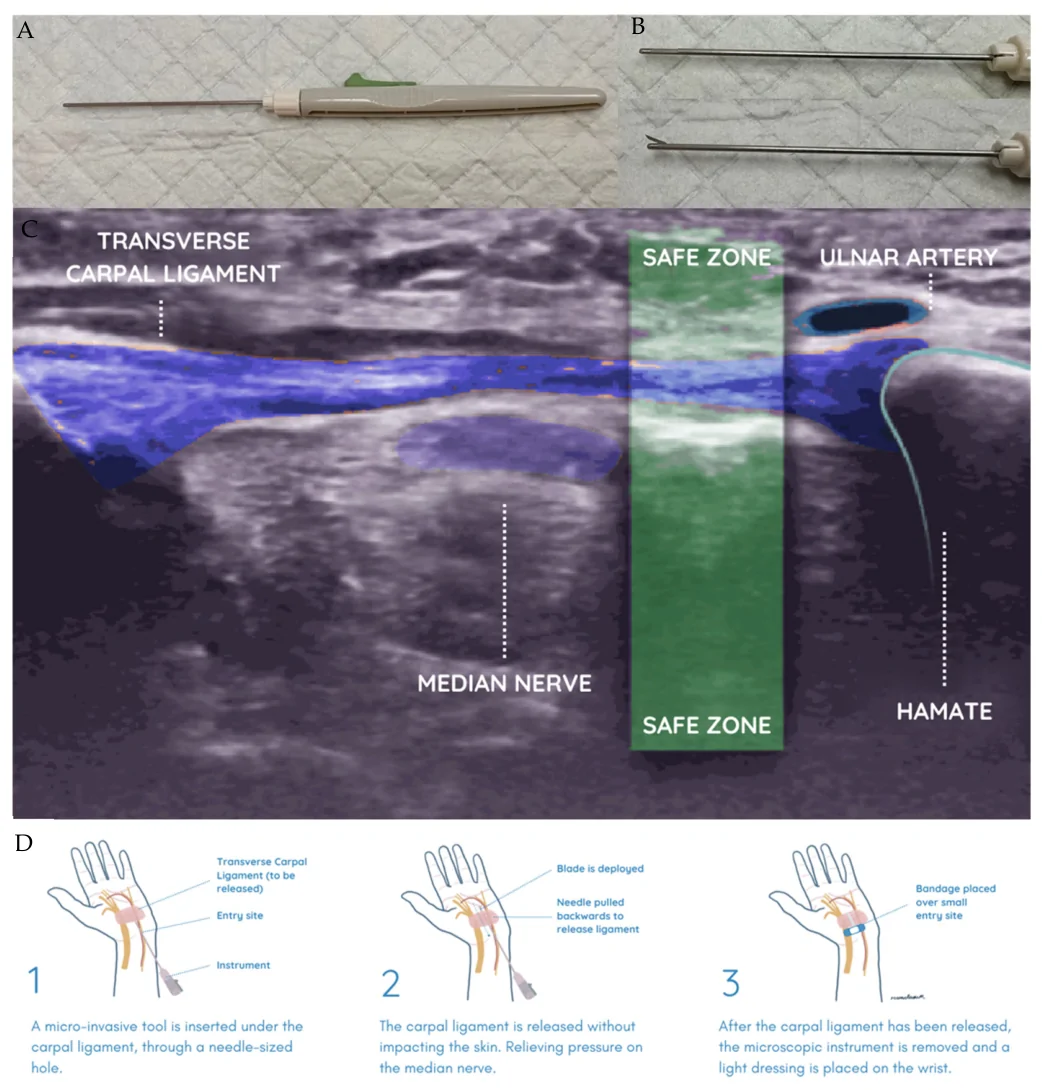
Representative images of the device and ultrasound-guided procedural planning used for Micro-CTR. (**A**) Full view of the retractable needle-mounted blade device used for office-based Micro-CTR in the un-deployed state. (**B**) Distal tip of the device demonstrating the blade in un-deployed and deployed positions. (**C**) Representative ultrasound image demonstrating anatomical mapping of the carpal tunnel, including the transverse carpal tunnel, median nerve, adjacent vascular structures, and the procedural safe zone prior to ligament division. (**D**) Simplified schematic illustrating key procedural steps of Micro-CTR. Images are provided for illustrative and educational purposes only and do not represent formal imaging outcome assessment.

Before ligament division, the carpal tunnel was mapped in short- and long-axis views. The transverse carpal ligament (TCL), median nerve, superficial palmar arch, ulnar vessels, flexor tendons, and relevant soft-tissue planes were identified. Doppler imaging was used to confirm vascular landmarks and support selection of a safe access trajectory. The TCL was assessed for thickness and continuity before decompression. Representative images of the device and ultrasound-guided procedural planning are shown in Figure 1.

Local anaesthesia was administered using 2% lidocaine under direct ultrasound guidance. Hydrodissection with 0.9% saline was then used to separate the TCL from the median nerve and develop a hypoechoic safety plane along the intended percutaneous access path. No tourniquet was used.

A single puncture access point (<2 mm) was created proximal to the carpal tunnel inlet. The microinvasive instrument was advanced under continuous in-plane ultrasound visualisation to traverse the TCL. The retractable blade was activated under real-time ultrasound guidance to divide the TCL along its ulnar aspect, with tissue separation and neurovascular safety assessed continuously (Figure 1). Completion of division was assessed dynamically under ultrasound [31,32,41].

Minor bleeding was managed with sterile gauze compression. The puncture site was covered with a simple sterile adhesive dressing, and no sutures were required. An adjustable wrist brace was provided for comfort and short-term protection for 24–48 h as required. Patients were discharged immediately after the procedure and advised to mobilise the hand freely from the day of surgery.

### 2.5. Operator Experience and Procedural Standardisation

All procedures were performed by credentialed clinician-operators experienced in ultrasound-guided carpal tunnel assessment and office-based Micro-CTR. All procedures followed a standardised protocol based on previously described ultrasound-guided microinvasive CTR techniques [30,31,41]. Procedural consistency was supported by standardised equipment, aseptic setup, ultrasound mapping, Doppler confirmation of vascular landmarks, local anaesthesia, hydrodissection, no-tourniquet technique, and post-procedure dressing and wrist brace protocols. Intraoperative documentation included device reference numbers, laterality, procedural time, technical variations, and ultrasound settings where applicable.

Because ultrasound-guided Micro-CTR is operator-dependent, operator experience and procedural standardisation are important contextual factors for interpreting reproducibility. Specific operator-level case-volume data and formal learning-curve outcomes were not collected as prespecified study variables; accordingly, the present study was not designed to evaluate learning-curve effects.

### 2.6. Data Collection and Instruments

#### 2.6.1. Patient-Reported Measures

Outcome data were collected using the Comprehensive Carpal Tunnel Recovery Questionnaire (CTRQ), derived from the Boston Carpal Tunnel Questionnaire (BCTQ) and supplemented with occupational recovery domains (Table 1). The questionnaire was administered preoperatively and postoperatively through voluntary patient completion.

No fixed postoperative assessment timepoints were mandated in order to comply with Human Research Ethics Committee approval and avoid coercive follow-up practices. The postoperative questionnaire was completed at a mean of approximately 3 weeks following the procedure. Participants were instructed to complete the questionnaire after recovery milestones had been reached or at a time of their choosing. Postoperative responses therefore reflected patient-reported recovery intervals rather than protocol-scheduled assessments.

Primary and secondary outcomes were based on self-reported number of days from surgery to RTW, return to driving, and return to household tasks, together with change in pain score on a 0–10 visual analogue scale (VAS) between preoperative and postoperative reporting. Because these outcomes were recorded as event-based intervals, variability in questionnaire reporting was not considered to materially affect estimates of recovery duration. Postoperative symptoms and events were captured using a structured multi-response checklist comprising swelling/haematoma, persistent numbness, pillar pain, and self-reported need for further treatment or revision, together with a dedicated free-text response field. For the primary postoperative-event analysis, participants were classified according to whether they selected at least one structured checklist category. Structured checklist categories were not mutually exclusive. Free-text responses were reviewed descriptively but were not reclassified into the structured event group because they were not collected through a standardised categorical measure. The questionnaire did not capture independent clinical adjudication, event severity, duration, management, or causal attribution. Accordingly, these findings are reported as patient-reported postoperative symptoms or events rather than clinically confirmed complications.

#### 2.6.2. Data Verification and Storage

Intraoperative documentation included device, reference numbers, procedural time, laterality, and any technical variations. All data were anonymised, stored in a secure database, and verified by a second investigator. Deidentified data were then stored on encrypted servers in accordance with Human Research Ethics Committee ethical protocol.

#### 2.6.3. Operative Materials and Instruments

All procedures were performed under ultrasound guidance using a sterile, single-use instrument set. The material inventory was standardised across all cases to ensure procedural consistency and traceability (Table 2). All instruments were sterile, single-use, and disposed of according to local clinical waste protocols. Procedural standardisation was documented in accordance with Hebbard et al. [31] and Ferreira-Silva et al. [32] to ensure reproducibility.

#### 2.6.4. Statistical Analysis

All analyses were performed in R version 4.5.2 (R Foundation for Statistical Computing, Vienna, Austria) and GraphPad Prism 11.0.0 (GraphPad Software, Boston, MA, USA). Continuous recovery outcomes were non-normally distributed and are therefore summarised as median and interquartile range (IQR). Analyses were conducted using available-case denominators, which are reported for each outcome. No imputation was performed.

The primary outcome was RTW time in days. Secondary outcomes were change in pain score from pre- to post-procedure on a 0–10 VAS, time to return to driving, time to return to household tasks, and duration of postoperative analgesic use.

For patients with paired pre- and postoperative pain scores, within-patient change was assessed using the Wilcoxon matched-pairs signed-rank test, and pain improvement is reported as median paired change with IQR.

For analyses by structured postoperative-event status, participants were classified according to whether they selected at least one postoperative-event category. Recovery outcomes were compared between participants with and without a structured self-reported postoperative event using the Wilcoxon rank-sum test. Free-text-only reports were summarised descriptively and were not incorporated into the primary group for comparison because they were not captured through a standardised categorical measure. Because three recovery endpoints were assessed in parallel (RTW, driving, and household tasks), Holm adjustment was applied to between-group *p*-values.

Time-to-event analyses were also performed for RTW, return to driving, and return to household tasks using the derived midpoint time variables and associated event indicators. Group differences by structured postoperative-event status were examined using log-rank tests and unadjusted Cox proportional hazards models. An HR below 1 indicates a slower rate of return.

An exploratory analysis was undertaken to characterise the influence of longer RTW intervals on the overall RTW distribution. Participants with both prolonged RTW, defined as ≥35 days, and at least one structured self-reported postoperative event were examined descriptively as a delayed-recovery subgroup. The ≥35-day threshold corresponded to the midpoint of the questionnaire category representing return at 4–6 weeks. This analysis was not used to redefine the primary RTW estimate or exclude participants from the main analysis. Fisher’s exact test, Wilcoxon rank-sum testing, and time-to-event analyses were used descriptively to compare this delayed-recovery subgroup with the remaining RTW responders.

Job category distribution was additionally compared using Fisher’s exact test to assess whether the prolonged RTW subgroup was explained by work type alone. Primary analyses are reported for the full cohort, with the sensitivity analysis presented as a robustness check.

Worker’s compensation status was reviewed descriptively but was not suitable for inferential modelling because variation was insufficient among RTW responders.

A two-sided *p*-value of <0.05 was considered statistically significant.

## 3. Results

### 3.1. Cohort Statistics and Data Completeness

The prospective cohort comprised 67 patients. Median age was 65 years (IQR 16.5). Outcome completeness was as follows: RTW, 48/67; return to driving, 65/67; return to household tasks, 65/67; painkiller use 64/67; and paired pre/post VAS pain scores, 66/67.

### 3.2. Overall Recovery Profile

**Figure 2:**
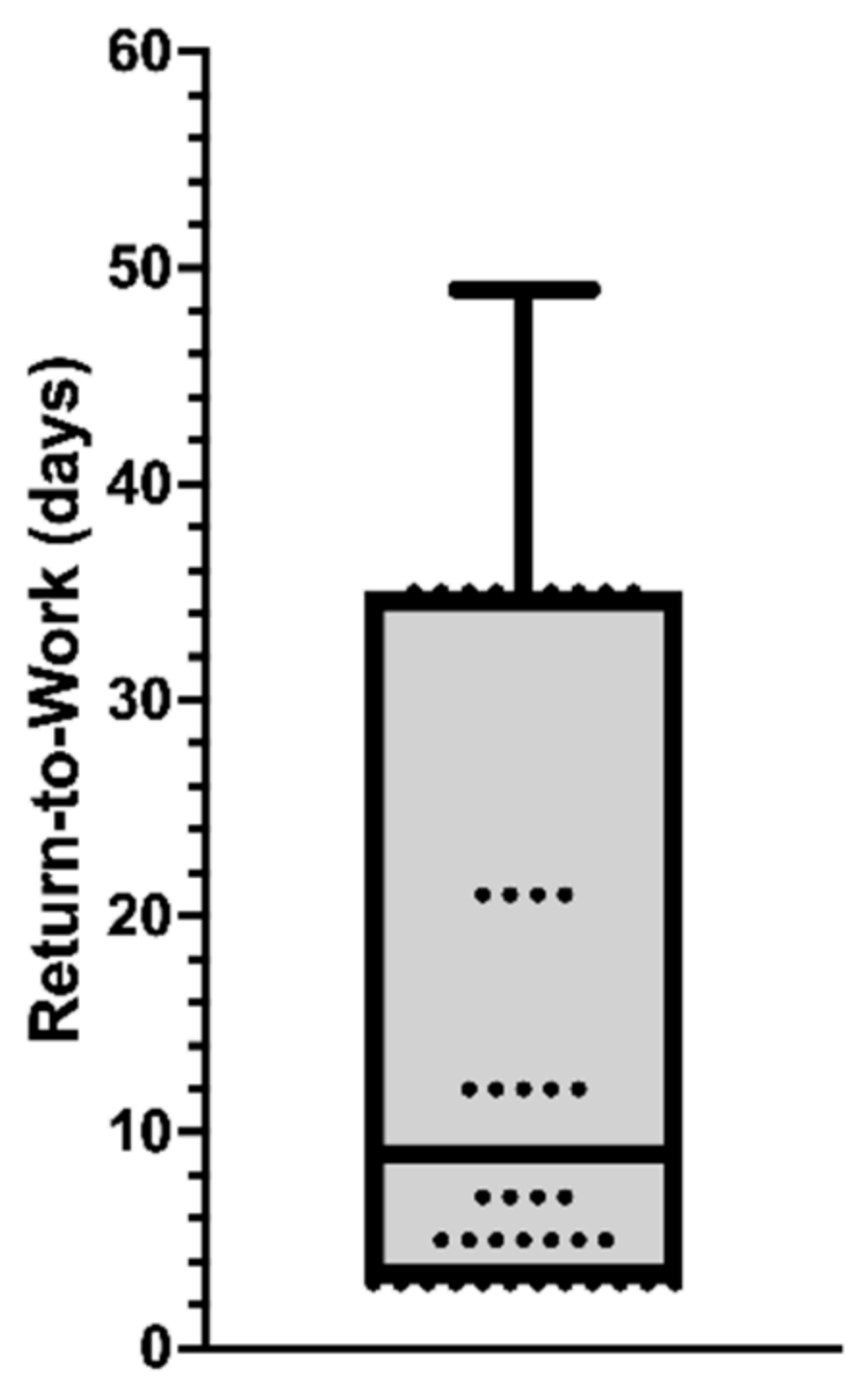
Distribution of Return-to-Work (RTW) times following microinvasive carpal tunnel release. Individual patient RTW intervals are shown with median and interquartile range, demonstrating a right-skewed distribution with a subgroup of delayed recovery.

Across the cohort, recovery milestones were generally rapid. Median time to return to driving was 5 days (IQR 6), median time to household tasks was 7 days (IQR 7), and median postoperative painkiller use was 2 days (IQR 3). Among patients with RTW data, median RTW was 9 days (IQR 30.5), although this outcome demonstrated substantial variability. The distribution of RTW times was right-skewed, with a majority of patients returning within the first two weeks and a smaller subgroup experiencing prolonged recovery (Figure 2).

### 3.3. Pain Outcomes

**Figure 3:**
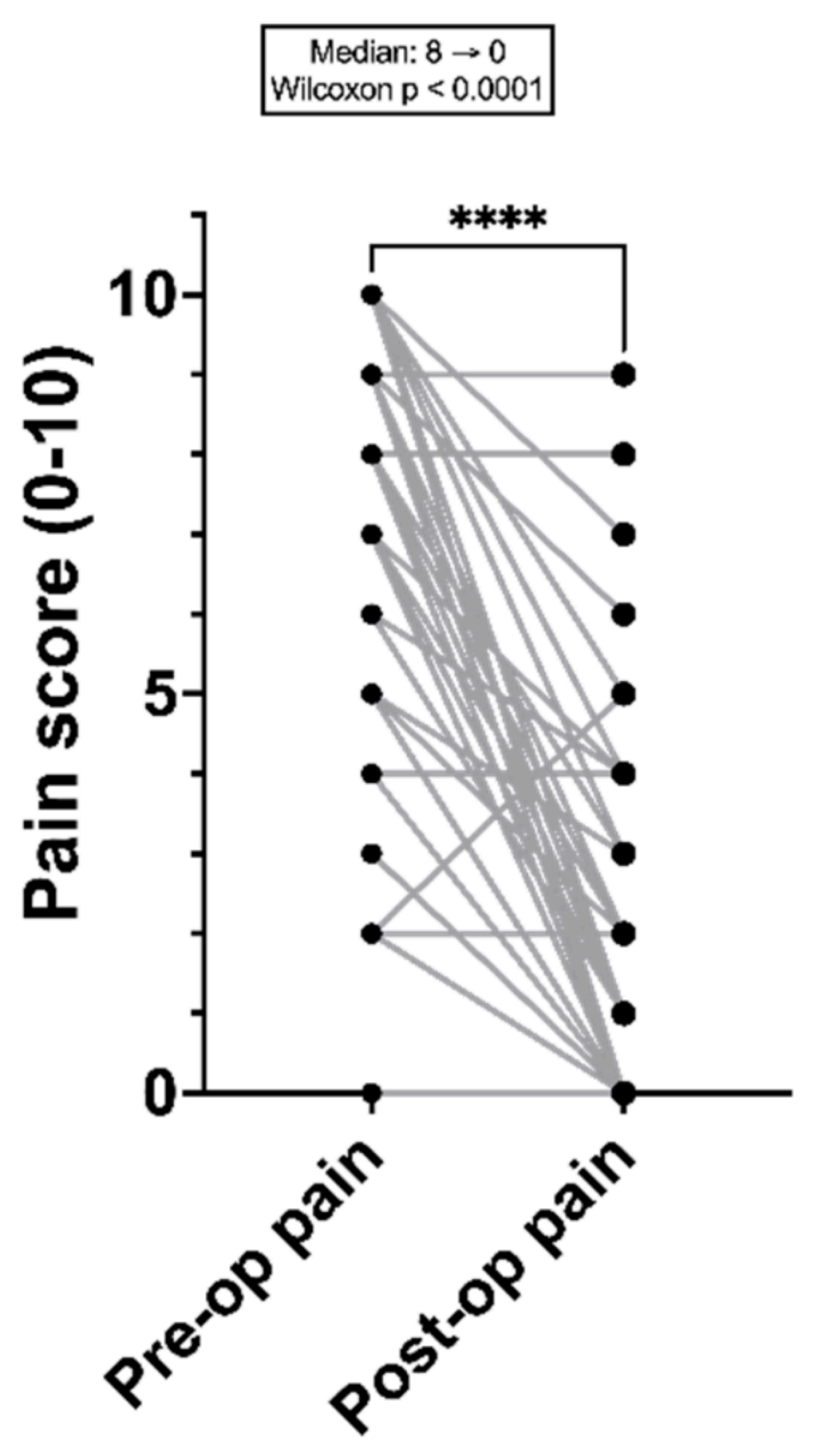
Change in patient-reported pain scores before and after microinvasive carpal tunnel release. Paired visual analogue scale (VAS) scores demonstrate substantial reduction in pain following the procedure. Each line represents an individual patient. **** *p* < 0.0001.

Among the 66 patients with paired VAS pain data, median preoperative pain was 8/10, compared with 0/10 postoperatively. The median improvement was 7 points (IQR 3). This reduction was statistically significant on Wilcoxon matched-pairs signed-rank testing (V = 1884.5, *p* = 1.38 × 10^−11^). Paired analysis demonstrated consistent within-patient reduction in pain (Figure 3).

### 3.4. Patient-Reported Postoperative Events and Recovery Time

On the structured postoperative questionnaire, 28 of 67 participants (41.8%) selected one or more postoperative-event categories, while 39 participants (58.2%) selected no structured event. Across the 28 reported events, 40 category selections were recorded. The most frequently selected categories were swelling/haematoma (*n* = 15), persistent numbness (*n* = 13), pillar pain (*n* = 7), and self-reported need for further treatment or revision (*n* = 5); categories were not mutually exclusive (Table 3). Structured checklist categories were not mutually exclusive; 40 categories selections were recorded across 28 participants. The seven free-text-only reports were summarised descriptively and were not included in primary structured-event comparison. These are patient-reported questionnaire findings and do not represent independently adjudicated clinical complication rates.

An additional seven participants who selected no structured event described a postoperative symptom or delayed recovery concern in the dedicated free-text field. These reports comprised numbness or tingling (*n* = 3), delayed recovery or reduced function (*n* = 2), persistent aching (*n* = 1), and morning stiffness (*n* = 1). These entries were summarised descriptively and were not incorporated into the primary structured-event comparison because they were not captured through a standardised categorical measure.

**Figure 4:**
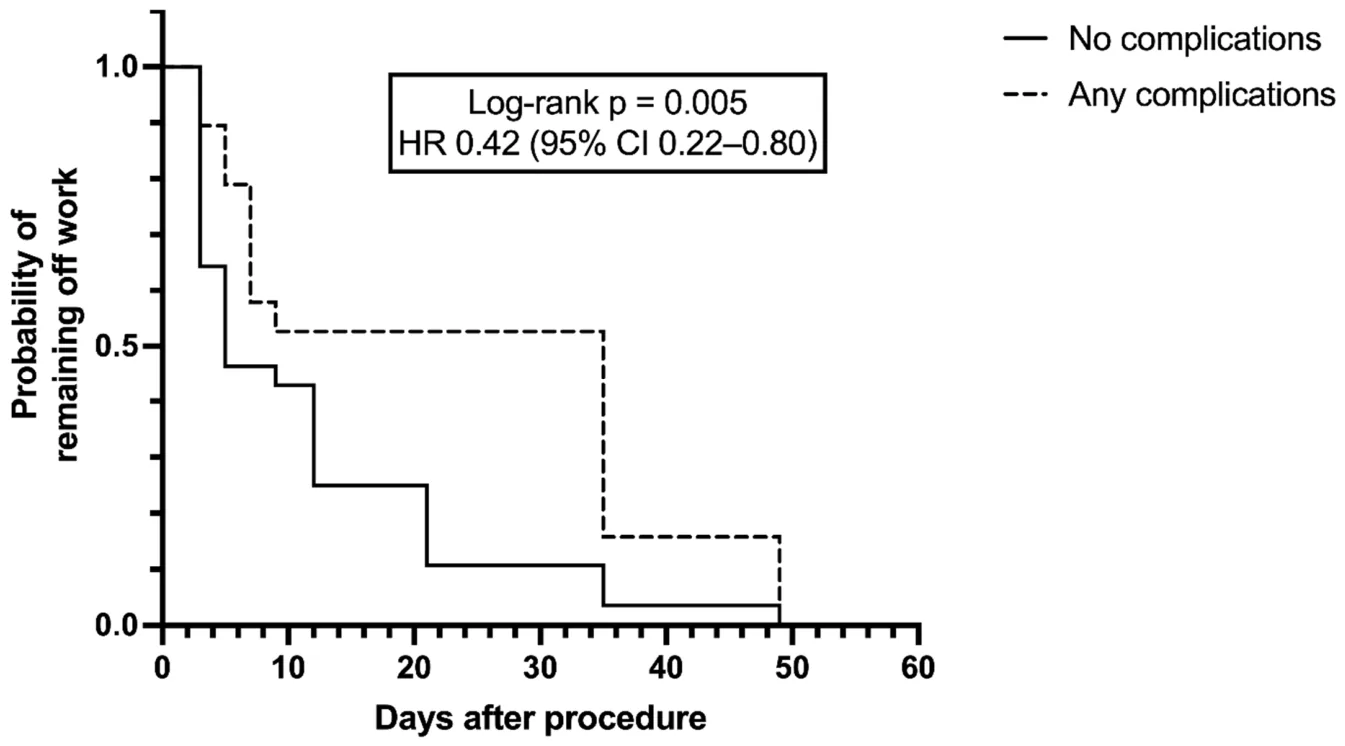
Kaplan–Meier analysis of Return-to-Work stratified by structured self-reported postoperative-event status. Participants selecting at least one structured postoperative-event category demonstrated a slower observed rate of RTW than those selecting no structured event (log-rank *p* = 0.005; HR 0.42, 95% CI 0.22–0.80).

Among participants with RTW data, 20 of 28 structured event reporters and 28 of 39 participants without a structured event contributed to analysis. Participants reporting at least one structured postoperative event had a median RTW of 35 days (IQR 28; *n* = 20), compared with 5 days (IQR 11.2; *n* =28) among those selecting no structured event. This difference was significant on Wilcoxon rank-sum testing (*p* = 0.00965) and remained significant following Holm adjustment (adjusted *p* = 0.0289). Time-to-event analysis similarly showed a slower observed rate of RTW among structured event reporters (log-rank *p* = 0.005; unadjusted HR 0.417, 95% CI 0.219–0.795; *p* = 0.00788) (Figure 4).

For return to driving (*n* = 65), median time was 5 days in both groups, with no significant difference on rank-sum testing (*p* = 0.493) or Cox analysis (HR 0.642, 95% CI 0.377–1.093; *p* = 0.102). For return to household tasks (*n* = 65), participants without a structured postoperative event returned at a median of 5 days (IQR 6; *n* = 37), compared with 9 days (IQR 16, *n* = 28) among structured event reporters. The unadjusted comparison was nominally significant (*p* = 0.0466), but this did not persist after Holm correction (adjusted *p* = 0.0932). Time-to-event analysis showed a slower observed rate of return among structured event reporters on log-rank testing (*p* = 0.03) and Cox regression HR 0.552 (95% CI 0.326–0.934; *p* = 0.0269).

These comparisons describe associations between questionnaire-reported postoperative events and recovery intervals. They do not establish a clinically adjudicated complication rate or a causal effect of individual postoperative events on RTW.

### 3.5. Exploratory Distributional Analysis of Prolonged RTW

An exploratory distributional analysis was performed to characterise the right-skewed RTW pattern observed in the full RTW responder cohort. Participants with both prolonged RTW, defined as ≥35 days, and at least one structured self-reported postoperative event were examined as a delayed-recovery subgroup. The ≥35-day threshold corresponded to the midpoint of the questionnaire response category representing return at 4–6 weeks and was used descriptively to evaluate the influence of longer recovery intervals on the overall RTW distribution.

**Figure 5:**
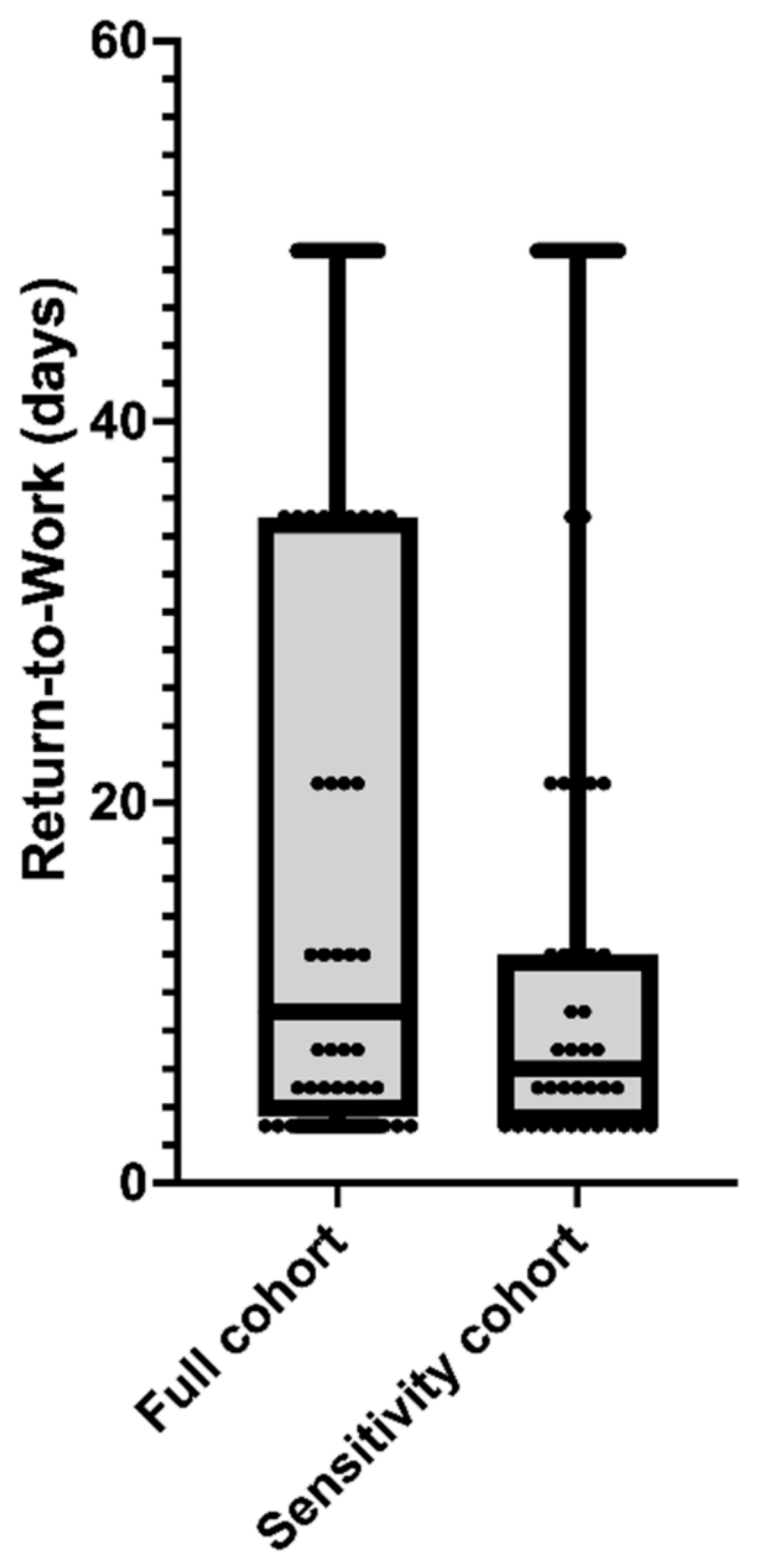
Exploratory comparison of RTW distributions in the full RTW responder cohort and a restrictive descriptive cohort excluding participants with both prolonged RTW (≥35 days) and at least one structured self-reported postoperative event. This analysis illustrates the influence of longer self-reported RTW intervals on the overall distribution. The full RTW responder cohort remains the primary analysis.

In the full RTW responder cohort, median RTW was 9 days (IQR 30.5). When participants with prolonged RTW and a structured self-reported postoperative event with both prolonged RTW and a structured self-reported postoperative event were removed for descriptive comparison only, median RTW was 5 days (IQR 9), while estimates for return to driving and household tasks were largely unchanged (Figure 5). Among RTW responders, prolonged RTW was associated with structured postoperative-event reporting (Fisher’s exact *p* = 0.001327; OR 9.61, 95% CI 1.96–66.24). Participants in the delayed-recovery subgroup had longer RTW intervals than the remaining RTW responders (Wilcoxon *p* = 3.262 × 10^−6^), and time-to-event analysis demonstrated a slower observed rate of RTW (log-rank *p* = 2 × 10^−6^; unadjusted HR 0.1627, 95% CI 0.0688–0.3848; *p* = 3.57 × 10^−5^). Job type distribution did not differ significantly between the delayed-recovery subgroup and the remaining RTW responders (Fisher *p* = 0.2129).

This analysis was not used to redefine the primary RTW estimate or exclude participants from the main analysis. Rather, it illustrates that the overall RTW distribution was influenced by a smaller group of participants with longer self-reported recovery intervals. Given that both postoperative-event status and RTW timing were questionnaire derived, these findings should be interpreted descriptively and should not be taken to establish a clinically adjudicated complication subgroup or causal relationship between individual events and delayed RTW.

### 3.6. Work-Related Variables

Worker’s compensation status was not suitable for formal modelling. In the full cohort, the responses were No = 37, Yes = 1, Missing = 29, and among RTW responders there were no workers’ compensation “Yes” responses, precluding meaningful inferential analysis. “Appropriate time off work” was variably reported and is best treated descriptively.

## 4. Discussion

In this prospective cohort study of office-based ultrasound-guided Micro-CTR, patients demonstrated rapid early patient-reported functional recovery, with a median of 9 days and early return to driving and household activities. Patient-reported pain scores also improved substantially, with a median reduction of 7 points on a 0–10 scale (Figure 3). These findings are consistent with the broader trend toward earlier functional recovery reported for less invasive CTR techniques, while remaining observational and non-comparative in nature [36].

The observed RTW distribution highlights the heterogeneity of recovery following Micro-CTR (Figure 2). While many participants returned to work within a short timeframe, RTW was right-skewed, with a smaller group reporting longer recovery intervals. This finding is important because summary recovery estimates alone do not fully capture the spread of postoperative recovery trajectories in office-based microinvasive practice.

Participants selecting at least one structured postoperative-event category reported longer RTW intervals than those selecting no structured event. Kaplan–Meier analysis further supported this distinction by demonstrating separation in RTW trajectories between participants selecting a structured postoperative-event category to those selecting no event (Figure 4). However, both postoperative-event status and RTW timing were questionnaire derived, and these findings should therefore be interpreted descriptively rather than causally. The association may reflect postoperative symptoms, perceived recovery, occupational demands, recall patterns, workplace expectations, or other unmeasured contextual factors.

The exploratory distributional analysis further demonstrated that longer self-reported RTW intervals had a substantial influence on the overall RTW distributions. This analysis was not used to redefine the primary recovery estimate or exclude delayed-recovery participants from the main analysis. Rather, it illustrates that early recovery after Micro-CTR is heterogeneous and that delayed RTW should be reported transparently alongside median recovery estimates.

These findings support the interpretation of RTW as a composite functional endpoint influenced by both postoperative course and occupational context. Although early recovery was observed in most patients, RTW is not determined solely by procedural factors. Workplace demands, compensation context, psychosocial factors, and postoperative complications may all contribute to variability in recovery timelines. This is particularly relevant when interpreting RTW across studies, where differences in patient populations and reporting methods may substantially influence reported recovery intervals.

The present findings should also be interpreted in the context of the existing literature. Our recent narrative review identified a general trend toward shorter RTW intervals with less invasive CTR techniques, while also highlighting considerable heterogeneity in reporting methods and outcome definitions [36]. The current study adds prospective cohort data using standardised patient-reported recovery measures and extends that literature by showing both rapid early recovery in many participants, while also transparently reporting delayed self-reported RTW intervals and their association with structured postoperative-event reporting.

The recovery variables examined in this study demonstrated right-skewed distributions, characterised by a majority of patients recovering within a short period and a smaller group reporting longer recovery intervals. Accordingly, these outcomes were summarised using medians and interquartile ranges and analysed using non-parametric and time-to-event methods. This approach provides a more representative description of recovery patterns in heterogeneous clinical data and avoids distortion of summary measures by a small number of prolonged recovery cases.

The technical advantages of ultrasound-guided percutaneous CTR should also be interpreted alongside several important procedural considerations. Ultrasound-guided release is operator-dependent and requires reliable visualisation of the TCL, median nerve, vascular structures, and adjacent soft tissues. Anatomical variants, suboptimal sonographic windows, or limited operator experience may affect procedural feasibility, safety, and completeness of release reported in some specimens despite avoidance of neural injury [29]. These considerations reinforce that device-specific techniques should not be treated as interchangeable, and that early patient-reported recovery does not by itself establish technical completeness, comparative superiority, or long-term durability.

From a clinical practice perspective, these findings suggest that early functional recovery after office-based ultrasound-guided Micro-CTR can be documented prospectively using patient-reported outcome measures that are relevant to everyday recovery. This is particularly important in emerging procedural settings, where transparent reporting of both typical recovery and delayed recovery subgroups may be more informative than headline summary statistics alone.

The present study was not designed to directly compare Micro-CTR with open, endoscopic, or other ultrasound-guided CTR techniques. Accordingly, no conclusions regarding comparative effectiveness, superiority, complication rates, or long-term durability between CTR modalities can be drawn. The findings should instead be interpreted as prospective, patient-reported early recovery data from a single office-based Micro-CTR cohort.

## 5. Conclusions

This study has several limitations. First, recovery milestones and pain scores were self-reported and collected without fixed postoperative assessment timepoints, reflecting real-world, patient-initiated reporting rather than protocol-mandated clinical review. Although this approach minimised coercion and preserved the voluntary nature of follow-up, it introduces potential recall bias, reporting variability, and response bias.

Second, postoperative symptoms and events were captured through questionnaire responses and were not independently clinically adjudicated. The study did not prospectively capture event severity, duration, management, objective confirmation, or causal attribution. Accordingly, the reported postoperative-event frequencies should not be interpreted as clinically confirmed complication rates.

Third, RTW data were available for 48 of 67 participants. Although available-case denominators were reported throughout and no imputation was performed, incomplete RTW reporting may have influenced recovery estimates. RTW is also affected by non-surgical factors, including job demands, workplace flexibility, compensation context, psychosocial factors, employer expectations, and patient preference. These factors were not comprehensively measured and may confound observed associations.

Fourth, the study was conducted at a single centre and involved an operator-dependent ultrasound-guided technique. Generalisability may therefore be limited by procedural expertise, ultrasound equipment, patient selection, local practice setting, and operator experience. The study was not designed to formally evaluate learning-curve effects.

Fifth, the follow-up period was short and focused on early functional recovery. The study did not assess medium- or long-term neurological recovery, recurrence, delayed complications, patient-reported symptom scores at later timepoints, or durability of decompression. Formal imaging outcome parameters, such as quantitative median nerve cross-sectional area or independent postoperative sonographic confirmation, were not included as research endpoints.

Finally, the observational design and absence of a control group preclude causal inference or conclusions regarding comparative effectiveness between CTR techniques. The findings should therefore be interpreted as prospective early recovery data from an office-based Micro-CTR cohort rather than evidence of superiority over open, endoscopic, or other ultrasound-guided approaches.

This single-centre prospective observational cohort describes early patient-reported recovery following office-based ultrasound-guided Micro-CTR. Among participants with available RTW data, median RTW was 9 days, with early return to driving and household activities and substantial patient-reported pain reduction over the early follow-up period.

Recovery was heterogeneous, with longer RTW intervals observed among participants reporting structured postoperative events.

These findings support the feasibility of using RTW and early functional milestones as pragmatic patient-reported outcomes in studies emerging CTR techniques. However, the study was descriptive, non-comparative, and limited by short follow-up, incomplete RTW data, and questionnaire-derived postoperative-event reporting. No conclusions regarding comparative effectiveness, superiority, clinically adjudicated complication rates, or long-term durability can be drawn.

## Figures and Tables

**Table 1:** Variables collected from the Comprehensive Carpal Tunnel Recovery Questionnaire.

Category	Variable	Scale	Description/Rationale
Demographic	Age, sex	Continuous/categorical	Descriptive baseline factors
Occupational	Job type (sedentary/light/heavy)	Ordinal	Physical demand predictor [11,34]
Clinical	Pain before and after Micro-CTR (VAS 0–10)	Continuous	Pain trajectory; predictor of RTW
Analgesic use	Days requiring pain medication	Continuous	Nociceptive recovery proxy [24]
Return-to-Work (RTW)	Days from procedure to resumption of employment	Continuous	Primary outcome metric [15,42]
Household activity resumption	Days to performing domestic tasks	Continuous	Functional independence proxy [4]
Compensation status	Yes/No	Binary	Socioeconomic modifier [11,43]
Satisfaction	4-point Likert (very satisfied → dissatisfied)	Ordinal	Global recovery perception [25]
Postoperative symptoms/events	Structured checklist and dedicated free-text response	Categorical/Free text	Patient-reported postoperative recovery concerns; not independently clinically adjudicated

**Table 2:** Materials used in a standard ultrasound-Guided Micro-CTR Procedure.

Category	Item (REF/LOT)	Manufacturer/Model	Purpose
Ultrasound System	Sonosite PX Portable Ultrasound System with High frequency L15-4 linear transducer (4–15 MHz)	FUJIFILM Sonosite Inc., Bothell, WA, USA	Real-time visualisation
Ultrasound interface	General Purpose Sterile Probe Cover Kit (ATRIS, REF 700-1022)	ATRIS Pty Ltd., Homebush, NSW, Australia	Sterile barrier for ultrasound transducer
Ultrasound coupling medium	Aquasonic^®^ 100 Ultrasound Transmission Gel	Parker Laboratories Inc., Fairfield, NJ, USA	Sterile acoustic coupling for optimised sonographic imaging and transducer contact
Primary cutting instrument	Retractable Needle-Mounted Blade (Smith & Nephew, REF 72174009)	Smith & Nephew, Inc., Andover, MA, USA	Microinvasive transection of the transverse carpal ligament under real-time US guidance
Accessory instruments	Blink Micro-Hook (REF HS945)	Blink Instruments, Solihull, West Midlands, UK	Tissue elevation and dissection under US visualisation
Drape and sterile field	Dr Peter Hebbard Carpal Tunnel Pack (MCA Medical, REF 2212)	MCA Medical Supplies Pty Ltd., Fairfield, NSW, Australia	Complete sterile drape kit for Micro-CTR
Local anaesthesia	Lidocaine HCl 2% (5 mL ampoules)	Pfizer Australia Pty Ltd./DBL, Sydney, NSW, Australia	Local infiltration for anaesthesia and hydrodissection
Hydrodissection/flushing	0.9% Sodium Chloride (10 mL ampoule)	Baxter Healthcare Pty Ltd., Old Toongabbie, NSW, Australia	Perineural plane hydrodissection
Needles/cannulae	BD Insyte 20G × 1.88” IV catheter; Smiths Medical Jelco 22G × 1”	BD/Smiths Medical International Ltd., Ashford, Kent, UK	Access creation for anaesthetic and hydrodissection
Sterile field consumables	Sterile Gauze Swabs (bpositive); Plastic Drape 120 × 90 cm (Multigate); Huck Towel 66 × 42 cm (Multigate)	Multigate/BPositive Healthcare Pty Ltd., South Yarra, VIC, Australia	Draping and wound care
Postoperative support	Adjustable Wrist Brace, Right (Success Co., A03346) Adjustable Wrist Brace, Left (Success Co., A03346)	Shenzhen Xiehang Technology Co., Ltd., Shenzhen, China	Post-procedure wrist bracing (as required)

**Table 3:** Patient-reported postoperative symptoms and events captured by questionnaire following Micro-CTR.

Questionnaire-Derived Category	*n*	% of Cohort (*n* = 67)
**Primary structured-event grouping**		
At least one structured postoperative event	28	41.8
No structured postoperative event selected	39	58.2
**Structured event categories**		
Swelling/haematoma	15	22.4
Persistent numbness	13	19.4
Pillar pain	7	10.4
Self-reported need for further treatment or revision	5	7.5
**Additional free-text only reports among checkbox-negative participants**		
Numbness or tingling	3	4.5
Delayed recovery or reduced function	2	3.0
Persistent aching	1	1.5
Morning stiffness	1	1.5
Any free-text-only symptom/event report	7	10.4

## Data Availability

The data presented in this study are not publicly available due to ethical and privacy restrictions relating to patient information. Deidentified data may be available from the corresponding author on reasonable request, subject to institutional and ethical approval.
